# Clinicians' perceptions of organizational readiness for change in the context of clinical information system projects: insights from two cross-sectional surveys

**DOI:** 10.1186/1748-5908-6-15

**Published:** 2011-02-28

**Authors:** Guy Paré, Claude Sicotte, Placide Poba-Nzaou, George Balouzakis

**Affiliations:** 1 Canada Research Chair in Information Technology in Health Care, HEC Montreal 3000 Cote-Ste-Catherine Road. Montreal, H3T 2A7, Quebec Canada; 2 Health Administration Department, Faculty of Medicine, University of Montreal, C.P. 6128 Downtown Station, Montreal, H3C 3J7, Quebec Canada; 3 FXinnovation, 400 Maisonneuve Blvd. West Montreal, H3A 1L4, Quebec Canada

## Abstract

**Background:**

The adoption and diffusion of clinical information systems has become one of the critical benchmarks for achieving several healthcare organizational reform priorities, including home care, primary care, and integrated care networks. However, these systems are often strongly resisted by the same community that is expected to benefit from their use. Prior research has found that early perceptions and beliefs play a central role in shaping future attitudes and behaviors such as negative rumors, lack of involvement, and resistance to change. In this line of research, this paper builds on the change management and information systems literature and identifies variables associated with clinicians' early perceptions of organizational readiness for change in the specific context of clinical information system projects.

**Methods:**

Two cross-sectional surveys were conducted to test our research model. First, a questionnaire was pretested and then distributed to the future users of a mobile computing technology in 11 home care organizations. The second study took place in a large teaching hospital that had approved a budget for the acquisition of an electronic medical records system. Data analysis was performed using partial least squares.

**Results:**

Scale items used in this study showed adequate psychometric properties. In Study 1, four of the hypothesized links in the research model were supported, with change appropriateness, organizational flexibility, vision clarity, and change efficacy explaining 75% of the variance in organizational readiness. In Study 2, four hypotheses were also supported, two of which differed from those supported in Study 1: the presence of an effective project champion and collective self-efficacy. In addition to these variables, vision clarity and change appropriateness also helped explain 75% of the variance in the dependent variable. Explanations for the similarities and differences observed in the two surveys are provided.

**Conclusions:**

Organizational readiness is arguably a key factor involved in clinicians' initial support for clinical information system initiatives. As healthcare organizations continue to invest in information technologies to improve quality and continuity of care and reduce costs, understanding the factors that influence organizational readiness for change represents an important avenue for future research.

## Background

The adoption and diffusion of clinical information systems (CIS) such as electronic medical record (EMR) systems, decision support systems, picture archiving and communication systems, and computerized provider order entry systems has become one of the critical benchmarks for achieving several healthcare organizational reform priorities, including home care, primary care, and integrated care networks [1-3]. Outcomes associated with the adoption of CIS in healthcare organizations include higher productivity levels among clinicians [4,5], better integrated care processes [6], and improved patient safety and quality of care [7,8], to name but a few.

However, these systems are often strongly resisted by the same community that is expected to benefit from their adoption and use. In some cases resistance has manifested itself in boycotts of installed computer-based systems [9,10] or threats of strikes by the medical staff to oppose the implementation of EMR systems [11,12]. In extreme cases, technological resistance induced the hospital management to remove state of the art CIS. For instance, Freudenheim [13] reports that physicians at the Cedars-Sinai Medical Center at Los Angeles rebelled against their newly installed computerized physician order entry (CPOE) system, complaining that the system was too great a distraction from their medical duties and forcing its withdrawal after it was already online in two-thirds of the 870-bed hospital. Nurses are also seen to be reluctant to use computers in areas closely related to patient care [14-16] for several reasons, such as the fear of being distracted or taken away from the patient and the lack of perceived alignment with nursing workflow/documentation processes [17]. Gillespie [18] reported that nursing resistance alone had caused the 'death' of several IT initiatives.

Prior research has found that favorable user attitudes are often associated with a high level of information technology (IT) adoption and acceptance [17,19-21]. In this regard, we argue that the early stages of the CIS project lifecycle deserve additional attention because early perceptions and beliefs play a central role in shaping future attitudes and behaviors such as negative rumors, involvement in the planning and design phases, and resistance to system usage. Furthermore, some authors concur that change management is most efficient when it is introduced at the earliest possible opportunity in the project lifecycle [22,23]. For these reasons, we decided to focus our attention on the pre-implementation stage, which is usually when change targets are introduced into the detailed project planning, the new system is seen or discussed for the first time, and initial impressions are formed about how work is likely to change [24,25].

Change targets' perceptions of the organization's readiness for change have been identified by change management theorists as one important factor in understanding potential sources of resistance [26-28]. An individual's perception of an organization's readiness for change is viewed as a concept similar to unfreezing, which is described as a process in which an individual's beliefs about pending change are influenced such that the imminent change comes to be seen as possible [29]. Readiness collectively reflects the extent to which individuals are cognitively and emotionally inclined to accept, embrace, and adopt a particular plan to purposefully alter the status quo [30]. These perceptions are conceptualized as existing on a continuum, from viewing the organization as capable of withstanding change and successfully adapting to it (high readiness for change) to believing the organization is not ready to undergo such a change (low readiness for change) [30].

While organizational readiness for change is an intuitively appealing construct, very few empirical studies in the health informatics field have focused on this phenomenon. The work of Snyder-Halpern [31-33] was all that could be found on the subject in the extant literature. In her studies, she defines organizational readiness very broadly as 'the level of fit between the IT innovation and the organization' and tests the hypothesis that a higher level of readiness leads to a lower level of innovation risk and a more successful CIS outcome [33]. The definition adopted by Snyder-Halpern is therefore more macro than the one used in this paper and applies to all phases of the CIS project life cycle. While the measure proposed by Snyder-Halpern serves as a proxy for the level of risk in a technological project, our measure is focused entirely on the notion of the ability to succeed at technological change as it is perceived by the users identified in the pre-implementation phase. We therefore see Snyder-Halpern's contribution as complementary to our own work.

The paper is structured as follows: First, we begin by reviewing relevant work in the change management and information systems fields that supports the hypothesized relationships between organizational readiness for change and its antecedents. Next, the paper describes the research design and the data that was collected in order to test our research model. This is followed by the presentation of the study results, their discussion, and concluding remarks.

### Research model

**Figure 1:**
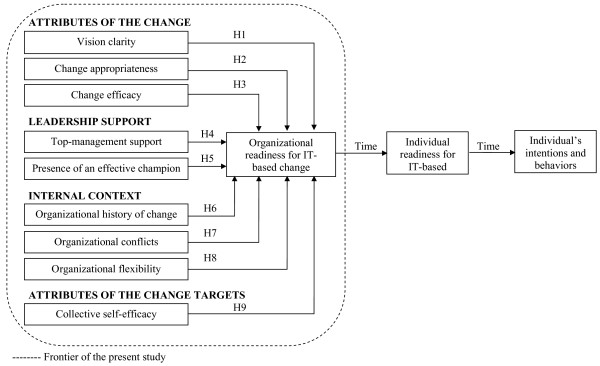
Research Model

The primary intent of this study was to investigate the variables associated with clinicians' perceptions of organizational readiness for change in the specific context of CIS projects. Based on Holt *et al.*'s [34] research model, four classes of variables (see Figure 1) were identified as possibly related to a clinician's interpretation of organizational readiness for change during the pre-implementation phase of CIS projects: the attributes of the change that is being introduced; the extent of leadership support for the proposed change; the organizational context where the change takes place; and the characteristics of the change targets. Each of these variables will be discussed.

### Attributes of the change

The attributes of the change refer to the 'what' factor of the change [34]. That is, one should first consider what is being changed. In most CIS projects, the change is not only associated with the new system, but also with local processes, organizational structure, roles and responsibilities, and compensation schemes [11]. As explained below, three attributes of the change are likely to have a significant influence on change recipients' perceptions of organizational readiness for change.

#### Vision clarity

Change management theorists posit that one of the key sentiments to creating change readiness is the sense that change is needed [26-28,35-37]. A clear vision provides much of the justification for such a sentiment. A discrepency beween current and desired performance helps legitimize the need for change. Otherwise, the motive for a change may be perceived as arbitrary [26]. The notion of vision clarity is also consistent with social accounts theory, which stipulates that information should be provided by change agents to explain why an organizational change is needed [38,39].

#### Change appropriateness

A second key sentiment emphasized by Armenakis *et al. *[26-28] is the sense that the change is appropriate. Indeed, in addition to believing that a change is needed, if employees are to support change, they must also believe that the specific change being proposed will effectively address the discrepancy. This sentiment is also consistent with social accounts theory [38] and is used to describe whether the proposed change is the correct one for the situation at hand. If the proposed change is viewed by employees as the incorrect approach for pursuing the vision, change targets may not be willing to 'buy-in' to the change or attempt to make it work [40]. Clearly, appropriateness of a change is important, because individuals may feel that some form of change is needed but may disagree with the specific change being proposed.

#### Change efficacy

A sense of efficacy, in the form of expectancy (efforts will lead to successful accomplishment), is a central tenet of most motivation theories [41]. To be motivated to support a change, individuals must not only feel that the change is appropriate but also that success is possible. In this sense, we believe that information from the environment may have a significant impact on individuals' perceptions of organizational readiness. If the proposed change has already been implemented successfully in similar organizations and this information has reached the appropriate individuals, one could conclude that they will see their organization as ready for a successful implementation. In contrast, if the press has reported prior failures in similar changes, one could expect some reticence on the part of the individuals affected by the change.

Based on this research, the following hypotheses are proposed:

Hypothesis one: Vision clarity will be positively related to perceived organizational readiness for change.

Hypothesis two: Change appropriateness will be positively related to perceived organizational readiness for change.

Hypothesis three: Change efficacy will be positively related to perceived organizational readiness for change.

### Leadership support

Social learning theory [42] posits that individuals sense through their interpersonal networks the support that exists throughout the organization. In this study, principal support describes the support from upper management as well as local change agents [28].

#### Top-management support

Many researchers have argued that senior managers play a crucial role in determining whether an information system project succeeds or fails [43-45]. Today, the need for strong leadership seems to be the generally accepted wisdom among information systems academics and managerial practitioners. When upper management is highly supportive of an IT project, greater resources are likely to be allocated to develop and support the new system [46], enhancing facilitating conditions [47] and, ultimately, increasing perceptions of organizational readiness.

#### Presence of a project champion

It has long been recognized by both practitioners and academics that it is highly risky to attempt complex change without a project champion [48,49]. In the IT context, champions are individuals who actively promote their personal vision for using IT, pushing the project over or around approval and implementation hurdles [50]. They may have initiated the process or been convinced of its necessity by other organizational members. Dong *et al. *[51] recently observed that perceived leadership behaviors of IT project champions exercise a direct and positive influence on users' attitudes toward the object of change. Their finding confirms the claim that project champions are effective leaders in tems of conveying visions and transcending users' self-interest for collective goals [50].

Extending this research, it is proposed that:

Hypothesis four: Top-management support will be positively related to perceived organizational readiness for change.

Hypothesis five: The presence of a project champion will be positively related to perceived organizational readiness for change.

### Organizational context

According to Holt *et al. *[34], internal context refers to the circumstances that describe the organization as it embarks on change. Mowday and Sutton [52] described internal context as the conditions external to change recipients that influence their beliefs, attitudes, intentions, and behavior. Prior research has led us to hypothesize that three organizational variables have a significant influence on change targets' perceptions of readiness.

#### Organizational history of change

To some degree, all organizations are idiosyncratic; that is, previous experiences have been stored in each organization in a pattern that makes the organization different from others that may on the surface appear very similar [53]. Organizations are dynamically evolving systems, and each has a history of resources, commitments, successes, and failures that shape the environment in which computer-based systems are developed and implemented [54]. Therefore, organizational history or memory might affect the way a change is framed in terms of previous initiatives undertaken by organization and hence have a great influence on the extent of IT implementation success.

#### Organizational conflicts

CIS implementation in healthcare organizations is characterized by social interactions. Among the many individuals and groups involved in the implementation process, there are usually managers, a project leader, a project champion, project team members, system developers, and a group of user representatives (clinicians). These actors have different interests and objectives for the adoption of a new CIS [55]. Hence, system implementation might be influenced by organizational politics and power relations [56,57]. Conflicting interests of different key actors and groups might lead to perceptions among targeted users that the organization is not ready for change.

#### Organizational flexibility

Some organizations are more agile and easily adaptable than others. For this reason, the degree to which organizational policies and practices are supportive of change may also be important to understanding how an employee perceives the organization's readiness for change [26]. Eby *et al. *[58] examined this issue in a study of two divisions of a national sales organization that was transitioning to work teams. Their results reveal that vendors' perceptions of their organization's ability to accommodate change by altering policies and procedures were strongly and positively related to perceived organizational readiness for change. Hence, we posit that clinicians are likely to hold unfavorable views about readiness for change when they perceive their healthcare organization's structure and policies as rigid and inflexible.

Based on prior research, we propose the following research hypotheses:

Hypothesis six: History of successful change experiences will be positively related to perceived organizational readiness for change.

Hypothesis seven: Organizational conflicts will be negatively related to perceived organizational readiness for change.

Hypothesis eight: Organizational flexibility will be positively related to perceived organizational readiness for change.

### Change targets' attributes

The fourth and final class of variables refers to the 'who,' or the organizational members who are required for change [34]. The variables are the attributes representing conditions internal to individuals that influence their beliefs, attitudes, and intention when confronted with change. In the present study, we focused on one of the most common individual factors that might influence perceptions of readiness, namely, individuals' skills or abilities.

#### Collective self-efficacy

Self-efficacy refers to sentiments of confidence in one's ability to succeed. This concept is included in Bandura's social learning theory [42], which suggests that employees who feel comfortable with their present skill set will believe that a different skill required to successfully execute the new job requirements can be mastered, such that they will be able to regain the comfort felt prior to the change. In this study we measure collective rather than individual efficacy, because the goal is to explain a construct at the organizational level. More specifically, we posit that individuals who perceive change targets, as a group, as capable of learning new work methods and tools will be more likely to look favorably on the organization's readiness for change.

Hypothesis nine: Collective self-efficacy will be positively related to perceived organizational readiness for change.

## Methods

To test the above hypotheses and increase the generalizability of our findings, two cross-sectional surveys were conducted. The first study investigated the pre-deployment of a mobile computing software solution in 11 ambulatory home care units. The second study took place in a large teaching hospital that had approved a budget for the implementation of an EMR system. Given the exploratory nature of this study, we favored a literal replication strategy where similar, not constrasting, results were predicted for each of the two CIS projects. The following paragraphs describe the pre-test and the two empirical studies.

### Pre-test and research settings

#### Pre-test

The study questionnaire was first pre-tested with five graduate students who are familiar with CIS as well as with four clinicians who had been involved in several CIS projects. Each respondent completed a first version of the questionnaire and provided feedback about the process and the measures, including the questionnaire administration time, and the clarity of the instructions and questions. The pretest indicated that the measurement instrument was relatively clear and easy to fill out. Following the pre-test, minor modifications were made to improve the wording of some items and the overall structure and presentation quality of the questionnaire.

##### Study one

A mobile computing project was carried out in an oncology and palliative care unit in Quebec, Canada in 2007. The core of the project was the implementation of a CIS that optimizes the process used to organize nursing activities taking place in patients' homes. The new SyMO package (Médisolution™) consists of a nursing care plan dictionary that covers all the procedures nurses need to perform in response to patient health problems, and an intervention plan module that allows nurses to create specific care plans for patients. Once she has arrived at the patient's home, the nurse uses the software to take notes on each procedure in the patient's care plan. This pilot project was the subject of an evaluative study that yielded encouraging results. Indeed, eight months after the implementation of the software application, the number of treated cancer patients increased by 6%, the average number of home visits by nurses increased by 0.7 visit per day, and the time allocated for direct patient care increased by 14% [5]. Given these positive results, senior administrators of Quebec's department of health and social services decided to invest additional funds in the project to verify whether the results were generalizable in the context of traditional home care services. The department asked 11 home care units in three different geographical regions to participate in the project. Study one investigated the pre-implementation phase of the mobile computing project in these ambulatory home care units.

The mobile computing project and the software package were officially presented to the nursing staff of each of the participating organizations at kick-off meetings held from May to November in 2009. The meetings were jointly organized by the managers of the health facilities and the supplier in order to present the scope of the project and its objectives, the roles and responsibilities of the key stakeholders, the planned deployment approach (including project phases), and the projected schedule and budget. The meetings also included a 60-minute demonstration of the software.

The data was collected at each of these meetings. Only nurses who would be affected by the change, *i.e.*, change recipients, were asked to stay in the room while the data were collected. Once the objectives of the study had been explained, a questionnaire was distributed to all the nurses in attendance. A total of 138 nurses completed the survey instrument, for a response rate of 90%.

##### Study two

As mentioned earlier, the second study took place in a large teaching hospital that had approved a budget for the acquisition of an EMR system. The institution, which specializes in the diagnosis and treatment of mental health illnesses, is divided into 10 clinical programs (*e.g.*, a mental health program for adults, a child psychiatry program, a geriatric psychiatry program, an eating disorders program). Each clinical program specializes in the care and treatment of various mental health illnesses. The staff is composed of approximately 400 clinicians (including mostly nursing staff and a balanced group of occupational therapists, social workers and psychologists), as well as 55 physicians.

The deployment of the EMR system represented a major organizational project that would affect the work processes and procedures across the entire hospital. The main objective was to maintain all patient information (admission, diagnosis, notes, prescriptions, test results, *et al*.) in a central patient database. At the time of the study, in early 2008, the healthcare organization was in the process of selecting a software vendor and an integrator. The project was presented at two staff meetings: the first meeting was for the entire nursing population, and the second was a general assembly that included all managers of the targeted clinical programs.

Data collection began shortly after the two official presentations. Over a period of three weeks, one of the researchers and the project manager visited the clinical staff at a weekly meeting in each of the 10 programs. At each visit, the local project manager summarized the key elements of the EMR project to staff and the researchers explained the objectives of the research project, addressing staff concerns and questions for approximately fifteen minutes. Then survey questionnaires were handed out, along with a pre-addressed return envelope. A total of 379 questionnaires were distributed to the clinicians. For physicians, direct email contact was initiated by the EMR project manager in a memorandum that presented the pending EMR implementation and the ongoing academic research project. A package was then mailed to each physician, containing a presentation letter, a copy of the questionnaire, as well as a pre-addressed postage-paid return envelope. The 55 physicians were asked to complete the survey within a week. Overall, a total of 235 questionnaires (207 from clinicians and 28 from physicians) were returned to the research team, for a response rate of 54%.

### Operationalization of variables and data analyses

Consistent with our research model, the survey's questions covered 10 variables. All except one were measured with four items. All the items were assessed on 7-point Likert scales ranging from strongly disagree to strongly agree. The items used to measure the dependent variable, namely, organizational readiness, were adapted from Eby *et al. *[58] and Rafferty and Simons [59]. As for the independent variables, vision clarity (VC) was measured using a scale adapted from Armenakis *et al. *[28]. Top-management support and change appropriateness were measured using scales adapted from Holt *et al. *[34]. Organizational flexibility was adapted from Rush *et al. *[60] and Eby *et al. *[58]. Group self-efficacy was measured using a scale adapted from Compeau and Higgins [61]. Finally, scales associated with change efficacy, organizational history of change, the presence of an effective project champion, and organizational conflicts were developed by the authors during a brainstorming session.

Scale items used to measure all study variables are presented in Appendix. Data analysis was performed using partial least squares (PLS), a structural equation modeling approach [62].

### Ethics approval

The present study was approved by the appropriate institutional ethics review boards.

## Results

### Sample profiles

As shown in Table 1, most participants in study one were women and had full-time positions. They were established registered nurses with an average of over 18 years of experience in the nursing profession and 10 years of seniority within their healthcare organization. The respondents' average experience with personal computers was 4.6 on a 7-point Likert scale where 1 is 'very unfamiliar with computers' and 7 is 'very familiar with computers.' In study two, one third of the respondents were men. More than half of the respondents (57%) were registered nurses and 12% were physicians. Respondents had over 15 years of experience in their profession and had spent, on average, 14 years in their current organization. Their level of experience with computers was similar to that of respondents in study one, with an average score of 4.8.

**Table 1:** Profile of respondents

		Study 1 (n = 134)	Study 2 (n = 237)
Gender	Male	2%	32%
	
	Female	98%	68%

Job status	Full time	80%	n/a
	
	Part time	20%	n/a

Job title	Registered nurse	100%	57%
	
	Social worker	-	9%
	
	Occupational therapist	-	4%
	
	Clinician (others)	-	19%
	
	Physicians	-	12%

Age	29 or less	14%	10%
	
	30 to 39	23%	21%
	
	40 to 49	35%	28%
	
	50 to 59	26%	34%
	
	60 and over	2%	7%

Level of computer experience (scale of 1 to 7)	Mean	4.6	4.8
	
	Standard deviation	1.4	1.5
	
	Minimum	1	1
	
	Maximum	7	7

Level of seniority within the organization (years)	Mean	9.8	14.0
	
	Standard deviation	6.1	10.5
	
	Minimum	<1	<1
	
	Maximum	35	38

Experience in the profession (years)	Mean	18.3	15.2
	
	Standard deviation	10.0	12.1
	
	Minimum	<1	<1
	
	Maximum	40	41

n/a = not available			

### Psychometric properties of the measures

Exploratory factor analyses of each reflective construct's items and their Cronbach alpha reliabilities were first examined as a check of unidimensionality. The results from these analyses revealed that all scale items associated with a given construct loaded highly (>0.60) on a single factor. Next, based on the results of the reliability analysis (Cronbach alpha), three items out of 39 were removed from their respective measurement instruments: OF4 (organizational flexibility), OC2 (organizational conflicts) and OHC4 (organizational history of changes). As a result, the remaining 36 items were then analyzed in PLS confirmatory factor analyses (CFA). Examination of revised construct reliabilities (Table 2), the variance shared between constructs (Table 3) and the cross-loadings (Tables 4 and 5) indicated that the psychometric properties of the 10 reflective constructs were acceptable. As can be seen, all Cronbach alphas were 0.71 or better and all item loadings were greater than 0.68.

**Table 2:** Reliability assessment of research model variables

	Final number of items	Cronbach alpha		Mean		Minimum		Maximum		Standard deviation	
		**Study one**	**Study two**	**Study one**	**Study two**	**Study one**	**Study two**	**Study one**	**Study two**	**Study one**	**Study two**

Vision clarity (VC)	4	0.79	0.88	5.9	5.2	2	1	7	7	1.0	1.3

Change appropriateness (CA)	4	0.90	0.92	5.9	5.1	1	1	7	7	1.1	1.4

Change efficacy (CE)	4	0.85	0.83	5.7	4.6	3	1	7	7	1.0	1.2

Top-management support (TMS)	4	0.76	0.81	5.3	4.9	2	2	7	7	1.1	1.3

Presence of a champion (C)	3	0.87	0.84	6.1	4.8	3	1	7	7	1.0	1.2

Organizational history of change (OHC)	3	0.79	0.76	5.1	4.6	3	1	7	7	1.0	1.1

Organizational conflicts (OC)	3	0.78	0.83	5.2	4.6	3	1	7	7	1.1	1.4

Organizational flexibility (OF)	3	0.71	0.75	4.4	3.9	1	1	7	7	1.1	1.2

Group self-efficacy (GSE)	4	0.82	0.84	4.4	4.1	1	1	7	7	1.2	1.4

Organizational readiness (OR)	4	0.89	0.88	5.7	5.0	2	1	7	7	1.1	1.3

**Table 3:** Variance shared between research model constructs

Study one	Variance									
	
	VC	CA	TMS	C	OHC	OC	OF	CE	GSE	OR
Vision clarity (VC)	**0.82**									

Change appropriateness (CA)	0.75**	**0.89**								

Top-management support (TMS)	0.42**	0.27*	**0.77**							

Presence of a champion (C)	0.48**	0.47**	0.67**	**0.89**						

Organizational history of change (OHC)	0.51**	0.50**	0.51**	0.55**	**0.79**					

Organizational conflicts (OC)	-0.25*	-0.26*	-0.55**	-0.50**	-0.45**	**0.77**				

Organizational flexibility (OF)	0.39**	0.49**	0.52**	0.50**	0.68**	-0.64**	**0.79**			

Change efficacy (CE)	0.60**	0.74**	0.59**	0.60**	0.66**	-0.47**	0.55**	**0.85**		

Group self-efficacy (GSE)	0.46**	0.54**	0.23*	0.34*	0.56**	-0.29**	0.51**	0.39*	**0.81**	

Organizational readiness (OR)	0.80**	0.80**	0.35*	0.45**	0.59**	-0.33**	0.60**	0.74**	0.51**	**0.88**

**Study two**	**Variance**									
	
	**VC**	**CA**	**TMS**	**C**	**OHC**	**OC**	**OF**	**CE**	**GSE**	**OR**

Vision clarity (VC)	**0.86**									

Change appropriateness (CA)	0.72**	**0.90**								

Top-management support (TMS)	0.44**	0.47**	**0.80**							

Presence of a champion (C)	0.67**	0.66**	0.73**	**0.87**						

Organizational history of change (OHC)	0.48**	0.51**	0.57**	0.60**	**0.76**					

Organizational conflicts (OC)	-0.11*	-0.13*	-0.32*	-0.30*	-0.47**	**0.81**				

Organizational flexibility (OF)	0.23**	0.25**	0.38**	0.38**	0.63**	-0.61**	**0.82**			

Change efficacy (CE)	0.71**	0.71**	0.43**	0.69**	0.57**	-0.18^ns^	0.28*	**0.82**		

Group self-efficacy (GSE)	0.20**	0.48**	0.41**	0.46*	0.61**	-0.31**	0.49**	0.49**	**0.82**	

Organizational readiness (OR)	0.79**	0.79**	0.51**	0.73**	0.60**	-0.29**	0.38**	0.72**	0.56**	**0.86**

** p < 0.001; * p < 0.05; ns = non significant

The bold numbers on the leading diagonal show the square root of the variance shared by the constructs and their measures. Off-diagonal elements are the correlations among constructs. For discriminant validity, diagonal elements should be larger than off-diagonal elements.

**Table 4:** PLS Construct cross-loadings of the research model (study one)

	1	2	3	4	5	6	7	8	9	10
VC1	**0.83**	0.72	0.67	0.54	0.70	0.34	0.32	0.57	0.66	0.70

VC2	**0.81**	0.69	0.54	0.57	0.67	0.26	0.31	0.52	0.45	0.74

VC3	**0.88**	0.75	0.57	0.42	0.63	0.30	0.47	0.43	0.51	0.72

VC4	**0.81**	0.71	0.68	0.51	0.65	0.36	0.42	0.69	0.51	0.76

CA1	0.71	**0.90**	0.55	0.69	0.53	0.41	0.41	0.60	0.49	0.80

CA2	0.70	**0.89**	0.59	0.71	0.54	0.49	0.43	0.63	0.43	0.81

CA3	0.69	**0.85**	0.57	0.68	0.71	0.59	0.47	0.66	0.58	0.79

CA4	0.71	**0.92**	0.60	0.64	0.62	0.43	0.42	0.62	0.57	0.83

TMS1	0.68	0.65	**0.79**	0.34	0.44	0.60	0.65	0.44	0.35	0.63

TMS2	0.57	0.53	**0.80**	0.41	0.36	0.53	0.69	0.49	0.36	0.57

TMS3	0.54	0.59	**0.84**	0.44	0.41	0.57	0.67	0.57	0.37	0.53

TMS4	0.59	0.52	**0.81**	0.39	0.45	0.54	0.61	0.51	0.46	0.55

C1	0.45	0.35	0.33	**0.87**	0.58	0.31	0.64	0.44	0.32	0.58

C2	0.36	0.44	0.32	**0.94**	0.60	0.27	0.65	0.36	0.38	0.52

C3	0.47	0.56	0.36	**0.88**	0.62	0.33	0.52	0.30	0.37	0.68

OHC1	0.57	0.48	0.51	0.67	**0.82**	0.60	0.26	0.61	0.53	0.55

OHC2	0.42	0.48	0.55	0.66	**0.87**	0.64	0.38	0.64	0.51	0.58

OHC3	0.49	0.52	0.67	0.64	**0.79**	0.59	0.30	0.66	0.59	0.64

OC1	0.30	0.68	0.65	0.70	0.56	**0.78**	0.50	0.51	0.66	0.62

OC3	0.45	0.65	0.59	0.68	0.54	**0.85**	0.54	0.42	0.62	0.66

OC4	0.41	0.59	0.59	0.59	0.47	**0.83**	0.58	0.48	0.60	0.57

OF1	0.60	0.46	0.44	0.44	0.66	0.41	**0.85**	0.55	0.49	0.70

OF2	0.58	0.45	0.49	0.43	0.59	0.25	**0.82**	0.53	0.59	0.73

OF3	0.47	0.55	0.34	0.50	0.69	0.34	**0.79**	0.49	0.57	0.76

CE1	0.61	0.45	0.55	0.33	0.71	0.65	0.71	**0.78**	0.31	0.78

CE2	0.58	0.56	0.59	0.56	0.67	0.68	0.67	**0.91**	0.47	0.77

CE3	0.49	0.49	0.66	0.57	0.66	0.50	0.62	**0.80**	0.45	0.79

CE4	0.36	0.54	0.50	0.48	0.67	0.60	0.63	**0.89**	0.51	0.71

GSE1	0.67	0.42	0.45	0.45	0.52	0.33	0.44	0.56	**0.86**	0.56

GSE2	0.58	0.55	0.46	0.43	0.60	0.35	0.58	0.43	**0.77**	0.65

GSE3	0.61	0.67	0.51	0.51	0.54	0.41	0.47	0.39	**0.88**	0.69

GSE4	0.53	0.64	0.34	0.50	0.59	0.43	0.51	0.47	**0.73**	0.61

OR1	0.71	0.70	0.54	0.73	0.58	0.42	0.71	0.69	0.67	**0.87**

OR2	0.69	0.62	0.32	0.71	0.54	0.35	0.73	0.62	0.69	**0.85**

OR3	0.73	0.66	0.56	73	0.52	0.46	0.75	0.70	0.54	**0.89**

OR4	0.72	0.58	0.45	0.71	0.47	0.34	0.73	0.70	0.60	**0.91**

**Table 5:** PLS construct cross-loadings of the research model (study two)

	1	2	3	4	5	6	7	8	9	10
VC1	**0.89**	0.70	0.59	0.53	0.74	0.31	0.29	0.51	0.69	0.68

VC2	**0.82**	0.65	0.52	0.54	0.61	0.29	0.30	0.57	0.44	0.70

VC3	**0.90**	0.72	0.50	0.40	0.66	0.32	0.49	0.41	0.56	0.73

VC4	**0.82**	0.69	0.58	0.52	0.62	0.32	0.44	0.67	0.53	0.71

CA1	0.68	**0.90**	0.54	0.68	0.52	0.44	0.47	0.62	0.46	0.66

CA2	0.72	**0.90**	0.56	0.73	0.51	0.48	0.43	0.61	0.47	0.73

CA3	0.61	**0.86**	0.56	0.66	0.70	0.57	0.49	0.57	0.59	0.76

CA4	0.70	**0.92**	0.65	0.63	0.60	0.44	0.39	0.65	0.58	0.72

TMS1	0.61	0.63	**0.68**	0.32	0.43	0.58	0.65	0.40	0.35	0.65

TMS2	0.52	0.51	**0.74**	0.46	0.39	0.50	0.62	0.48	0.32	0.59

TMS3	0.55	0.55	**0.69**	0.42	0.47	0.52	0.60	0.56	0.40	0.51

TMS4	0.51	0.54	**0.74**	0.32	0.42	0.57	0.60	0.58	0.43	0.59

C1	0.42	0.36	0.38	**0.85**	0.59	0.30	0.61	0.51	0.34	0.57

C2	0.32	0.47	0.33	**0.90**	0.58	0.29	0.61	0.35	0.39	0.55

C3	0.47	0.51	0.39	**0.85**	0.53	0.35	0.49	0.31	0.35	0.60

OHC1	0.56	0.42	0.53	0.69	**0.80**	0.62	0.31	0.56	0.59	0.50

OHC2	0.41	0.47	0.58	0.62	**0.80**	0.63	0.39	0.59	0.48	0.59

OHC3	0.45	0.55	0.61	0.64	**0.72**	0.58	0.33	0.58	0.52	0.61

OC1	0.32	0.59	0.62	0.68	0.57	**0.77**	0.53	0.49	0.61	0.64

OC3	0.42	0.61	0.56	0.62	0.56	**0.86**	0.53	0.40	0.69	0.67

OC4	0.47	0.52	0.55	0.62	0.41	**0.87**	0.54	0.49	0.60	0.54

OF1	0.58	0.42	0.46	0.40	0.67	0.44	**0.81**	0.52	0.46	0.71

OF2	0.59	0.41	0.48	0.42	0.60	0.29	**0.88**	0.56	0.57	0.70

OF3	0.46	0.54	0.33	0.49	0.68	0.33	**0.75**	0.49	0.54	0.74

CE1	0.59	0.42	0.54	0.31	0.70	0.67	0.72	**0.74**	0.35	0.72

CE2	0.56	0.55	0.61	0.54	0.66	0.61	0.62	**0.70**	0.49	0.61

CE3	0.42	0.44	0.60	0.57	0.67	0.56	0.63	**0.72**	0.44	0.68

CE4	0.32	0.52	0.52	0.44	0.62	0.61	0.65	**0.69**	0.52	0.60

GSE1	0.66	0.41	0.49	0.50	0.49	0.30	0.49	0.54	**0.85**	0.55

GSE2	0.56	0.59	0.41	0.41	0.57	0.32	0.59	0.47	**0.83**	0.63

GSE3	0.58	0.62	0.53	0.52	0.57	0.42	0.49	0.38	**0.87**	0.66

GSE4	0.51	0.65	0.32	0.51	0.57	0.44	0.49	0.43	**0.71**	0.59

OR1	0.70	0.72	0.51	0.74	0.63	0.41	0.73	0.67	0.62	**0.89**

OR2	0.66	0.67	0.34	0.70	0.53	0.38	0.70	0.64	0.65	**0.84**

OR3	0.74	0.67	0.52	0.71	0.54	0.48	0.71	0.66	0.57	**0.78**

OR4	0.75	0.60	0.42	0.69	0.44	0.33	0.68	0.63	0.62	**0.89**

Two criteria that are recommended for assessing discriminant validity are a square root of average variance extracted (AVE) that is higher than inter-construct correlations and indicators loading more highly on their corresponding factor than on other factors [63,64]. The results shown in Table 3 indicate that diagonal elements (AVE) were higher than off-diagonal elements (inter-construct correlations). For their part, the cross-loadings in Table 4 and Table 5 show that all indicators loaded more highly on their own factor than on other factors. Overall, these findings indicate that the measurement model has satisfied the recommended convergent and discriminant validity criteria.

### Hypothesis testing

Table 6 presents the PLS path coefficients along with the proportion of explained variance in the dependent variable. In study one, four of the hypothesized links in the research model were supported, with change appropriateness (H2), organizational flexibility (H8), vision clarity (H1), and change efficacy (H3) explaining 75% of the variance in organizational readiness. On the other hand, five hypotheses were not supported. More specifically, top-management support (H4), presence of an effective champion (H5), organizational history of change (H6), organizational conflicts (H7), and collective self-efficacy (H9) were not found to be associated with the dependent variable. In study two, four hypotheses were also supported, two of which differed from those supported in study one: the presence of an effective champion (H5) and collective self-efficacy (H9). In addition to these variables, vision clarity (H1) and change appropriateness (H2) also helped explain 75% of the variance in organizational readiness. Five hypotheses were not supported in study 2: change efficacy (H3), top-management support (H4), organizational history of change (H6), organizational conflicts (H7), and organizational flexibility (H8).

**Table 6:** PLS Results

	Path coefficients	
	
	Study one (n = 138)	Study two (n = 235)
Vision clarity (VC)	0.18*	0.27**

Change appropriateness (CA)	0.46***	0.25**

Change efficacy (CE)	0.17*	0.06

Top-management support (TMS)	0.07	0.03

Presence of a project champion (C)	0.05	0.23**

Organizational history of change (OHC)	0.07	0.07

Organizational conflicts (OC)	0.01	0.09

Organizational flexibility (OF)	0.21**	0.01

Group self-efficacy (GSE)	0.02	0.16*

**% of variance explained in the dependent variable**	**0.75**	**0.75**

*** p < 0.001; ** p < 0.05; * p < 0.01

## Discussion

The purpose of this study was to identify variables associated with clinicians' perceptions of organizational readiness for change in the particular context of CIS projects. Change management theorists argue that there are four classes of antecedents that have a direct effect on perceived organizational readiness for change: the attributes of the change that is being introduced, the extent of leadership support for the proposed change, the organizational context where the change is being implemented, and the characteristics of the change targets. As explained in the preceding section, our analyses suggest adequate reliability as well as convergent and discriminant validity of the measurement instruments used in this study.

Our findings imply that CIS project managers and leaders would benefit from explicitly addressing change content perceptions (change attributes) when pre-implementing CIS in healthcare organizations. More specifically, the results of this study indicate that two types of change sentiments -- vision clarity and change appropriateness -- have a significant and positive influence on clinicians' perceptions of organizational readiness for CIS-based change. In other words, our results support the idea that CIS projects have greater chances of success with a compelling reason, *i.e.*, a reason that makes change targets recognize and accept that a change is needed (vision clarity). In addition to believing that change is needed, if change targets are to support the CIS project, they must also believe that the specific change being proposed is the correct one in the present context (change appropriateness). Change theorists also argue that in order to be motivated to support a change, individuals must not only feel that the change is appropriate but also that success is possible. In this regard, sources of information outside the organization can be used to bolster messages sent by the change agents. This is effectively what happened in the CIS project reported in study one. Indeed, the success of the pilot project carried out in the oncology and palliative care unit in 2007 was highly publicized through newspaper and magazine stories, as well as on television at the start of 2008. In the spring of 2008, the project was nominated for an award in the annual 3M innovation contest organized by Quebec's professional order of nurses. The publicity surrounding the project had a significant, positive effect on the perceptions of nurses in the 11 units of their organization's capacity to successfully implement the proposed change. The effect of this variable (change efficacy) was not supported by study two. One possible explanation may be that a system had not yet been selected at the time the data was collected, and the hospital concerned was one of the first health facilities in Quebec to deploy an EMR system. This meant that little information was available in the media about this type of project when the readiness in this facility was measured.

Second, we hypothesized that leadership support would be positively associated with organizational readiness for change. For one thing, it is important to ask why top-management support was not associated with organizational readiness. One explanation may be the speed with which CIS projects were launched. In both studies, the project announcement came suddenly, only a few weeks before the survey. It was only as the project was being officially presented -- at the same time as data collection -- that most of the targeted clinicians were informed of management's support for the project. The second variable, the presence of an effective project champion, was supported only by study two. One possible explanation may be tied to whether or not someone had been identified to assume this role at the time that we measured organizational readiness. Even though this role had been filled in each of the 11 facilities in study one, no champion had yet been identified in the 10 hospital clinics participating in the project in study two. Not knowing who would assume this role in the project may have exacerbated the uncertainty experienced by respondents, such that they perceived this variable as very important to the project's success.

Third, our findings provided minimal support for hypotheses related to the organizational context within which change is implemented. More specifically, we observed that an organizational history of change and the political climate in the organization were not supported as indicators of readiness for change. In study one, only clinicians' perceptions of their organization's ability to accommodate changing conditions by altering policies and procedures were strongly related to perceived readiness for change. As mentioned above, some organizations are more adaptable and flexible than others. As such, regardless of change targets' comfort level with the nature of a CIS project, if the organization's structure is perceived to be inflexible and rigid, it appears that targeted clinicians are likely to hold less favorable attitudes about the organization's readiness for change. This finding was not, however, supported in study two. One possible reason is that study two was conducted in a single health facility, as compared to the 11 facilities in study one, which presented varying levels of flexibility.

Finally, collective self-efficacy was found to be positively related to organizational readiness for change only in study two. This finding might also depend on the timing of the organizational readiness assessment. A software provider (and package) had already been selected in study one, while in study two the technology represented a relatively abstract concept to the respondents because organizational readiness measurement took place prior to the call-for-tender process. The nurses in each of the 11 facilities in study one had already attended a demonstration of the software when they completed the questionnaire, and this may have reassured them about their collective ability to learn and use their future work tool. This was not the case for study two respondents who only had a vague idea of what the functionalities of the EMR system would be.

## Limitations

This study is not without certain methodological limitations that should be considered when interpreting the results. First, the data were collected using a single, self-reported questionnaire. When self-reports are used, concerns often arise as to whether common method bias is responsible for the observed relationships. Second, our analyses were based on a single type of technology (CIS) and a single group of change recipients (healthcare professionals), which limits the generalizability of our findings. However, our study was conducted in 11 ambulatory care organizations (study one) and 10 clinical units at a large teaching hospital (study two) to ensure a certain variety in terms of context. Third, the research design used in this study also presents limitations inasmuch as it did not allow us to assess clinicians' changing perceptions of their organization's readiness for change over time. For instance, while we believe that the presence of an effective project champion influences change targets' perceptions, the champion's actions and commitment might be more influential during the subsequent implementation phase when he or she drives consensus and manages resistance to change. In a similar way, as the project progresses toward the implementation phase, leadership behaviors exercised by upper management (*e.g.*, a clarifying vision, allocating the required financial and human resources to the project) are likely to play a greater role in the change process. Even though the argument may be difficult to support in the case of the organizational history of change, we believe that organizational conflicts and politics as well as group self-efficacy will prove to play major roles in the implementation phase; hence the importance of carrying out longitudinal studies and making a clear distinction between the pre-implementation and the implementation phases.

## Conclusions

Some authors have argued that the management of IT-based organizational change needs to begin as early as possible. The present study represents an initial attempt at understanding the variables that affect clinicians' perceived organizational readiness for change by suggesting that vision clarity and change appropriateness, as well as change efficacy, organizational flexibility, the presence of an effective champion, and collective self-efficacy, are all important antecedents.

Our findings have several implications for both practice and research. In practical terms, conducting a pre-implementation readiness assessment will help CIS project managers and decision makers choose whether they should initiate such a project or implement less costly, preliminary steps that will prepare the organization for the anticipated change. In this light, it is interesting to note that two of the 11 sites that participated in study one have not deployed the software package because of low readiness scores. As for future research, we believe that our results raise two important issues. First, more studies are needed in order to confirm which determinants are most significant in terms of perceived organizational readiness for CIS-based change. It would also be interesting to verify which antecedents are likely to emerge, based on the particular context of the project, and those that have an impact on the perceptions of change targets, independent of the context. Second, future research should investigate the extent to which organizational readiness is predictive of successful CIS adoption. Prior studies have also revealed that perceived organizational readiness significantly influences an individual's readiness for change [58,59,65] which, in turn, is a precursor of individual adoption or resistance behaviors (see Figure 1). It would therefore be important to have an analysis of the link between the level of perceived organizational readiness and clinicians' individual readiness for CIS-based change. Third, other key predictors could be included in the research model to further increase its explanatory power. For instance, clinicians' early perceptions of the usability of the technology *per se *[66] may also play a significant role in predicting clinicians' early perceptions of organizational readiness. In short, as healthcare organizations continue to invest in CIS to enhance quality and continuity of care, understanding the factors that contribute to an effective change process represents an important avenue for continued research.

## Competing interests

The authors declare that they have no competing interests.

## Authors' contributions

GP and CS participated in the design of the study and the development of the measurement instrument, carried out data collection in study one, performed the statistical analyses, and they were responsible for the writing of the manuscript. GB participated in the literature review and the development of the measurement instrument and he was responsible for collecting data in study two. PB contributed to the literature search and was involved in drafting the manuscript and revising it critically for important intellectual content. All authors read and approved the final manuscript.

## Appendix

### Questionnaire items (as framed in study one)

#### Vision Clarity (VC)

VC1. I believe there are legitimate reasons for us to introduce a new computer-based system in our unit.

VC2. We definitely need new tools to improve the way we work around here.

VC3. There are a number of rational reasons for the deployment of a new information system in our unit.

VC4. A new computer-based system is needed to improve our clinical processes.

#### Change Appropriateness (CA)

CA1. I think that nurses in our unit will benefit from the use of SyMO.

CA2. The deployment of SyMO will contribute to our unit's overall performance.

CA3. The deployment of SyMO matches the priorities of our unit.

CA4. The implementation of SyMO will prove to be best for our unit.

#### Change Efficacy (CE)

CE1. I know nurses outside our unit who had successful experiences with SyMO.

CE2. SyMO has been successfully deployed in clinical units similar to ours.

CE3. SyMO has received positive reviews in the press (*e.g.*, newspapers, magazines, newsletters, *et al.*).

CE4. I believe the provincial movement toward the electronic medical record represents a driving force for the deployment of SyMO in our unit.

#### Top-Management Support (TMS)

TMS1. Managers in our unit are committed to the deployment of SyMO.

TMS2. Managers in our unit have stressed the importance of this change.

TMS3. Managers have sent a clear message that the deployment of SyMO will occur in our unit.

TMS4. Nurses have been encouraged to embrace the upcoming deployment of SyMO.

#### Champion (C)

C1. There is a champion who actively promotes the deployment of SyMO in our unit.

C2. The SyMO project has a credible and trustworthy champion.

C3. There is a champion who will be able to push the SyMO project over or around implementation hurdles.

#### Organizational History of Change (OHC)

OHC1. Our unit has successfully implemented other technological changes in recent years.

OHC2. Nursing staff in our unit have had negative experiences with technological projects in the past (reversed item).

OHC3. Our unit is usually successful when it undertakes all types of changes.

OHC4. Information technology initiatives have been encouraged and are common practices in our unit (removed item).

#### Organizational Conflicts and Politics (OCP)

OCP1. Mutual trust and cooperation among nursing staff in our unit is strong (reversed item).

OCP2. Recent attempts to change the way we work in our unit have been hindered by political forces or conditions (removed item).

OCP3. The climate in our unit is mainly characterized by conflicts and disputes.

OCP4. Staff frustration is common in our unit.

#### Organizational Flexibility (OF)

OF1. Our unit is structured to allow superiors to make changes quickly.

OF2. It is easy to change procedures in our unit to meet new conditions.

OF3. Getting anything changed in our unit is a long, time-consuming process.

OF4. Policies and procedures in our unit allow us to take on new challenges effectively (removed item).

#### Group Self-Efficacy (GSE)

SE1. All nurses in our unit are highly computer literate.

SE2. It won't take a long time before nurses in our unit feel comfortable using SyMO.

SE3. Using a computer effectively is no problem for the nursing staff in our unit.

SE4. In general, nursing staff in our unit have low computer skills (reversed item).

#### Organizational Readiness (OR)

OR1. I believe SyMO can be successfully implemented in our unit.

OR2. Managers should delay the deployment of SyMO in our unit (reversed item).

OR3. The deployment of SyMO in our unit is timely.

OR4. Our unit is ready to take on this technological change.
